# Everybody's Page

**Published:** 1892-06-11

**Authors:** 


### Everybody's Page.

The only respectable thing about drunkenness is its antiquity. In this matter even our much-vaunted British Constitution has to, we will not say to give away, but to take an inferior position.

It is a common delusion that beer being brewed from malt contains all the nourishing properties of the barley which yields the malt. As a matter of fact, these important properties are converted in the process of malting into sugar, which only maintains the warmth of the body and supports respiration, and into alcohol, the tendency of which is to make the body colder and to destroy healthy structures.

A new departure has taken place in baby farming, and the project has for its patron no less a personage than M. Zola. It is to be very different from the establishments which have earned for themselves such an evil reputation, and a theatrical performance has already been given in Paris at the Vaudeville for its benefit. The object for which this daring enterprise has been started is stated to be the rescue of infants from the stupidity of nurses. Therefore, we suppose only specially trained nurses will be employed in the "Pouponnière," for so it is called, the career of which will be watched with interest.

The success of travelling libraries in districts which do not and for good reasons cannot, possess public libraries has exceeded the hopes of their most sanguine supporters. The manner in which they are appreciated is in itself evidence of the eagerness of all classes to seek after knowledge and is such as to lead to representations being made in Oxfordshire to the County Council in favour of establishing a county lending library. An additional spur has been given to the movement by the application in this direction of Mr. T. H. Green's gifts to the funds of the Oxford University Extension system.

The Lord Chancellor has recently raised a warning voice against "learned ignorance." Though his words were directed against the marvellous amount of misinformation now passing current with regard to religious questions, they can be applied with justice in a much more general sense. There has never been a time, owing, doubtless, to the spread of so-called education, when "learned ignorance" has been more widespread. The magnificent education which was to have been placed within reach of the humblest and the poorest has not turned out all that was promised for it, and most young people, victimised by a faulty system, have a smattering of all things and know nothing well. Someone has said of our Universities, that whatever else they don't do, they at least enable a young man to write a magazine article. What a glorious result of the most expensive training in the world!

A friend of ours, on returning home a few evenings ago, found his manservant hopelessly drunk. Though he expected friends to dinner that night he promptly dismissed the incapable "Jeames." A few mornings after this episode he received a letter from the erring soul expressing disgust at the treatment received, which was "just the kind to make men Anarchists!" His reasoning on the subject would have been nearer the mark had it led him to the conclusion that drink is more likely to make a man an Anarchist than the just treatment he received.

Simplicity and refinement are the essential characteristics of life in Japan. The houses, which are spacious, are constructed without foundations. Light wooden uprights resting on flat stones support the thatched or tiled roof. The walls, both outside and those which divide the rooms, are formed of latticed panels which slide over one another, or can be removed altogether if desired. These panels are filled with translucent paper. At night the house is closed in with wooden shutters. The rooms, which are raised about a foot above the ground, are covered with soft padded matting kept spotlessly clean. In the centre of the living room is a shallow square pit lined with metal and filled with charcoal, for the purposes of cooking and warming, or the rooms are warmed with moveable metal braziers. There is no furniture present, no chairs, tables, beds, chests of drawers, pictures, or knick-knacks. The matted floor serves alike for chairs, table and bed. To keep it absolutely clean all boots, shoes, and sandals are left on the ground outside. In one corner of the room is the raised recess or Tokonoma, where a vase containing a branch of a flowering shrub, and a hanging picture or kakemono is always found. The absence of furniture means the absence of many cares, and as two wooden chopsticks and small lacquer bowls serve for all the purposes of eating, there is no need for plate, glass, knives, forks, spoons, dinner services, and table linen. Thus life is simplified, though it loses, at the same time, none of its refinement, for no people can be more dainty and particular in their food, more neat and beautiful in dress, and more courteous and self-restrained in manner than the Japanese. From babyhood all are trained to sit on the soles of their feet, and this is with the Japanese the attitude of repose. To most Europeans it is of extreme discomfort, and induces distressing "pins and needles" in a very short time. Kneeling thus on the floor, all work is done, and at night time the padded quilts or futons are spread on the matting, and with one quilt beneath and another above, sleep can be enjoyed as comfortably as in bed. Before the evening meal is taken it is the invariable custom throughout Japan for every member of the household to take a dip in the family bath, which is heated to a temperature of 110 deg. to 120 deg., at which heat it is found to be very refreshing.

